# Why dignity is a troubling concept for AI ethics

**DOI:** 10.1016/j.patter.2025.101207

**Published:** 2025-03-14

**Authors:** Jon Rueda, Txetxu Ausín, Mark Coeckelbergh, Juan Ignacio del Valle, Francisco Lara, Belén Liedo, Joan Llorca Albareda, Heidi Mertes, Robert Ranisch, Vera Lúcia Raposo, Bernd C. Stahl, Murilo Vilaça, Íñigo de Miguel

**Affiliations:** 1 President’s Cabinet and Ethics in Research Department, Spanish National Research Council, Madrid, Spain; 2 Institute of Philosophy, Spanish National Research Council, Madrid, Spain; 3 Department of Philosophy, University of Vienna, Vienna, Austria; 4 Department of Philosophy 1, University of Granada, Granada, Spain; 5 Department of Philosophy and Society, Complutense University of Madrid, Madrid, Spain; 6 Department of Philosophy and Moral Sciences, Ghent University, Ghent, Belgium; 7 Department of Public Health and Primary Care, Ghent University, Ghent, Belgium; 8 Faculty of Health Sciences Brandenburg, University of Potsdam, Potsdam, Germany; 9 NOVA School of Law, Universidade Nova de Lisboa, Lisboa, Portugal; 10 CEDIS - Research & Development Centre on Law and Society, NOVA School of Law, Campus de Campolide, Lisbon; 11 School of Computer Science, University of Nottingham, Nottingham, UK; 12 Fundação Oswaldo Cruz/Escola Nacional de Saúde Pública, Rio de Janeiro, Brasil; 13 Ikerbasque, Basque Foundation for Science, Bilbao, Spain; 14 Department of Public Law and Chair in Law and Human Genome, University of the Basque Country, Leioa, Spain

## Abstract

The concept of dignity is proliferating in ethical, legal, and policy discussions of AI, yet dignity is an elusive concept with multiple philosophical interpretations. The authors argue that the unspecific and uncritical employment of the notion of dignity can be counterproductive for AI ethics.

## Main text

Dignity is an important concept in ethics and human rights advocacy. Not surprisingly, dignity has been employed prominently in recent AI regulation, institutional declarations, and policy instruments on machine learning. As a current example, the term dignity appears six times in the AI Act of the EU (Regulation (EU) 2024/1689), a comprehensive legal framework on AI that presents dignity both as a value and a right, but without being explicitly defined. It also appears in top-tier policy documents, such as the recent UN report “Governing AI for Humanity,” where dignity appears as an AI-related risk linked to a long string of concerns, including “manipulation, deception, nudging, sentencing, exploitation, discrimination, equal treatment, prosecution, surveillance, loss of human autonomy and AI-assisted targeting”.^1^ Similarly, dignity has been identified in widely cited reviews as one of the most repeated principles in AI ethics guidelines,^2^^,^^3^ although it often remained undefined.^2^

However, the use and abuse of dignity raises deep philosophical questions about its meaning, normative justification, and usefulness in applied debates. The utility of this notion was formerly challenged in related disciplines such as bioethics.^4^^,^^5^ In previous works, some of us debunked the employment of dignity as a convincing argument to stop developments in human genetic technologies.^6^^,^^7^ In AI ethics, moreover, the concept of dignity has been used to defend contradictory positions—something that can arguably happen with other ethical principles. Dignity has been used *both* to defend and to oppose the development of care robots for the elderly.^8^^,^^9^ Similarly, the adoption of sex robots may recognize the dignity of the sexual life of persons with disabilities, but it may at the same time infringe on the dignity of women.^10^ More problematically, one of us previously denounced that dignity is sometimes used as a mere slogan in ethical discussions on AI.^10^ Given these problems, what role (if any) should dignity play in ethical disputes about AI?

In this article, we make a public call to question the unspecific use of dignity and to rethink its function in ethical, legal, and policy discussions of AI. We begin by clarifying the main conceptions of this normative notion. We then briefly show AI’s ambivalent impact regarding dignity and object inflammatory uses that loosely posit AI as a threat to human dignity. We next address another largely neglected complexity in this debate, showing how the term dignity is gaining traction in debates about the moral status of AI entities. Finally, we conclude by recapitulating our arguments and offering some closing thoughts.

### The many conceptual faces of dignity

The concept of dignity has a multifaceted nature. Part of the problem of using this notion in ethical discussions of AI derives from the multiple meanings of the term. It is therefore useful to clarify some of its main interpretations. We shall divide these conceptions into status dignity and intrinsic dignity.^10^^,^^11^

On the one hand, status dignity depends on specific roles or behaviors and is not attributable to all people. There are three subtypes of status dignity—also sometimes called “aspirational dignity.”^10^^,^^11^ First, the historically original sense of dignity was aristocratic and referred to the exhibited quality of a person acting in conformity with her superior rank. Second, comportment dignity is attributed to those people who behave following proper manners and certain social expectations of politeness. Third, meritorious dignity is attributed to those individuals who act virtuously and with a high sense of self-worth. These three types of status dignity conceive dignity as a variable quality that distinguishes people from each other.

On the other hand, intrinsic dignity is mostly understood as an inviolable property of humans. Here, human dignity refers to the high level of moral value of humans just for being humans. This conception of dignity as an inviolable attribute of human beings comes mainly from two sources. On the one hand, the Christian tradition confers dignity to humans by the mere fact that they are created in the image and likeness of God. On the other hand, the Kantian tradition has popularized the concept of dignity as an inviolable intrinsic value of any human being. According to Kant, dignity stems from human rational nature and moral autonomy (the capacity to be morally self-legislative, i.e., to give moral rules to oneself), and which obliges us to treat beings with dignity as ends in themselves and never as mere means. This conception of human dignity as an inviolable worth has become a prevalent legal concept in the wake of the atrocities that took place during the Second World War, underlying the justificatory ground of universal human rights and fundamental rights in several legal frameworks.^9^

### The impact of AI on human dignity

The concern that AI can threaten human dignity has been widely discussed regarding many applications, such as lethal autonomous weapons, sex robots, care robots, or automated decision-making systems. For example, the threat of AI-based autonomous weapons systems to human dignity has played a starring role.^12^ While there are undoubtedly multiple ethical reasons to argue against the development of killer robots, it is striking why the dignity argument has been so prominent.

To begin with, it is often unclear to what dimension of dignity critics are referring when they say that AI in general—or autonomous weapons in particular—jeopardizes human dignity. On the one hand, it makes little sense that AI could endanger the human dignity of the combatants, conceived as an intrinsic quality of human beings, since, in this conception, dignity is an inviolable attribute. Strictly speaking, this conception of dignity as intrinsic worth is unconditional and cannot be degraded. Thus, it is unclear how AI can menace the *inviolable* dignity intrinsic to human life. More charitably, however, when people denounce a violation of human dignity, what they most likely mean is that someone was treated *as if* they did not possess human dignity. That is, although unconditional dignity was still there, it was just ignored. On the other hand, status dignity does account for degradation and humiliation, since it depends on features that are not attributable to all people, so it would make more sense to say that AI may impact dignity from this viewpoint. But, in this second conception (which is relational and at worst hierarchical), the harm caused by AI must be specified to assess its impact on status dignity. All in all, the argument that AI poses a threat to human dignity is not self-evident and would require more detail for a minimally satisfactory use.

We should rethink this dignity talk for two further reasons. Firstly, the rhetoric of dignity often comes devoid of ethical reasons. Since it elicits strong emotional responses against concrete technological uses, it becomes a common strategy with campaigning advantages.^12^ Precisely, it was this inflationary usage of dignity—as a categorical claim to rule out concrete technological applications—that led to the concept of dignity being labeled as a “conversation stopper” that was not conducive to rational argumentation in bioethics.^5^ This is why the human dignity argument sometimes works as a mere slogan in AI ethics.^10^

Secondly, the argument is problematic because it suffers from generality and sometimes functions as a proxy for other ethical reasons. When the human dignity argument is used in debating AI, it often surreptitiously appeals to other, more concrete values or ethical arguments. Frequently, evocations of human dignity seek to protect equality between people, non-discrimination, human autonomy, or avoidance of undue harm.^4^ Other times, dignity is invoked as a substitute for other arguments such as the risk of objectification (treating a person as lacking autonomy) or the risk of dehumanization (loss of human presence in a particular practice).^9^ Thus, given that dignity sometimes functions as a proxy for other ethical claims, it makes sense that this normative concept has been employed in contradictory ways in AI ethics debates, as mentioned in the introduction. And while some have argued that this terminological flexibility is a reason for its conceptual adoption,^9^ we believe that it is more fruitful for AI ethics debates to use the most concrete arguments possible and ideally avoid ambivalent generalizations.

### The dawn of machine dignity debate

As if the philosophical query about human dignity were not difficult enough, recent contributions are probing the notion of machine dignity or robotic dignity. The machine dignity debate addresses whether non-human AI-based entities could possess the properties that underlie the ascription of the special moral status we call dignity. Could the increasing agent-like nature of AI challenge the anthropocentric framing of inviolable dignity? The philosophical debate is divided.

Krämer argued that the term dignity is best to be avoided in the context of robot ethics, since it brings more confusion than clarity to the debate on the moral status of AI.^13^ Other authors, by contrast, have argued that dignity can be consistently ascribed to technological entities. J.S. Gordon evaluated the rationalist-Kantian notion of dignity and showed that AI could have this kind of dignity if it achieves certain properties. For this reason, he rejected those arguments that deny the potential moral rights of robots based on human dignity.^14^ Mosakas elaborates on Gordon’s arguments and shows that the substrate and origin of a certain entity do not determine its dignity^15^; so, for both authors, the moral status that underlies the concept of dignity need not be exclusive to human beings.

These contributions show that the debate on the dignity of AI is likely to increase in the coming years. Although we are agnostic about whether the use of the concept of dignity enhances or hinders the debate about the moral status of AI entities, it is easy to foresee that its use will generate misgivings. Human dignity—when conceived as an intrinsic attribute—is built on the view of human exceptionalism, according to which human beings are essentially different and superior to other species and therefore deserve special consideration and respect. That assumption has unsurprisingly faced numerous criticisms. More importantly, future research in animal cognition and even AI may reinforce objections to the human exceptionalism that underlies the view of dignity as an inviolable moral status unique to our species. Perhaps in the distant future, if we wish to continue to use the term dignity, we must stop attributing it only to members of our species.

### Concluding remarks

We have argued that dignity is an overly complex concept to be used loosely in AI ethics. Dignity is open to different conceptualizations, so an unspecific use of this notion is undesirable. Likewise, dignity is sometimes used as a mere slogan and for inflammatory purposes to oppose certain technological developments in a way that is not necessarily rational. In other situations, furthermore, appeals to dignity are used as proxies for other ethical arguments. And debates about the moral status of AI may increasingly co-opt the concept, perhaps forcing us to rethink the foundations of human dignity.

Other reasons may merit more attention in the future. Especially, is dignity indispensable for the development of a global approach to AI ethics? While human dignity is an important bedrock of the Universal Declaration of Human Rights, the concept of dignity has clear roots in Western philosophy and Christian tradition.^10^ There is cultural variation in the interpretations of human dignity too.^12^ So, in future contributions, the potential of dignity as a universal category for global AI ethics should be reconsidered.

In closing, wording choice is not an aseptic decision in AI ethics. Progress in ethical discussions often requires conceptual progress. To be sure, ethical terms are open to various conceptualizations and there may be various legitimate uses. But vaguely using complex terms that have multiple nuances, such as dignity, may do a disservice to AI ethics.

## Acknowledgments

J.R.'s work is funded by the European Commission – NextGenerationEU, through Momentum CSIC Programme: Develop Your Digital Talent. J.R., J.I.d.V., F.L., and J.L.A. thank the funding of the project AutAI (grant PID2022-137953OB-I00), supported by the State Research Agency of the Spanish Government (MICIU/AEI/10.13039/501100011033) and ERDF, EU. T.A. thanks the funding of TAILOR: Training the next generation of researchers that will bring rehab robots to clinical practice HORIZON-MSCA Doctoral Networks program (grant agreement 101168724) and empoderDAT: Empoderamiento del paciente, espacio de datos de salud e inteligencia artificial como pilares del nuevo sistema sanitario Fundación Séneca-Agencia de Ciencia y Tecnología de la Región de Murcia (grant number 22041/PI/22). B.L. and T.A. acknowledge funding from Spanish Ministry of Science and Innovation through project VAE Value-Awareness Engineering TED2021-131295B-C31. J.L.A. has also benefited from a research contract FPU (FPU22/03178), funded by the Spanish Ministry of Science. H.M. has received funding from the European Research Council (ERC) under the European Union’s Horizon 2020 research and innovation programme (grant agreement no. 919841—DIME). R.R. thanks Digital Medical Ethics Network (DiMEN) funded by VolkswagenStiftung (grant number 9B233). B.C.S. was supported by the Engineering and Physical Sciences Research Council (Horizon Digital Economy Research ‘Trusted Data Driven Products’: EP/T022493/1) and (AI UK: Creating an International Ecosystem for Responsible AI Research and Innovation: EP/Y009800/1), the European Union (STRATEGIC project, GA no. 101145644), and by UK Research and Innovation (UKRI) under the UK government’s Horizon Europe Guarantee funding scheme (10123282). M.V. thanks the Conselho Nacional de Desenvolvimento Científico e Tecnológico (CNPq): APQ/PRÓ-HUMANIDADES (421523/2022-0); Chamada Universal (421419/2023-7); Bolsista de Produtividade em Pesquisa - PQ2 (315804/2023-8). I.d.M. acknowledges the support of the Basque Government, Ayudas a grupos de investigación, Grupo de Investigación, IT 1541-22, and research project GODAS, PID2022-137140OB-I00, funded by MCIN/AEI/10.13039/501100011033/FEDER, UE. We also thank the audience of Rio de Janeiro for their insightful comments on a previous presentation of this work.

## Declaration of interests

The authors declare that the research was conducted in the absence of any commercial or financial relationships that could be construed as a potential conflict of interest.
